# Targeted next-generation sequencing of circulating free DNA enables non-invasive tumor detection in myxoid liposarcomas

**DOI:** 10.1186/s12943-022-01523-x

**Published:** 2022-02-14

**Authors:** A. E. Eisenhardt, A. Schmid, J. Esser, Z. Brugger, U. Lausch, J. Kiefer, M. Braig, A. Runkel, J. Wehrle, R. Claus, P. Bronsert, A. Leithner, B. Liegl-Atzwanger, J. Zeller, R. Papini, M. von Laffert, B. M. Pfitzner, G. Koulaxouzidis, R. E. Giunta, S. U. Eisenhardt, David Braig

**Affiliations:** 1 Department of Plastic and Hand Surgery, Medical Center - University of Freiburg, Faculty of Medicine, University of Freiburg, Hugstetter Str. 55, 79106 Freiburg im Breisgau, Germany; 2 Department of Radiology, Medical Physics, Medical Center - University of Freiburg, Faculty of Medicine, University of Freiburg, Freiburg im Breisgau, Germany; 3 Minerva Imaging, Lyshøjvej 21, 3650 Ølstykke, Denmark; 4 Department of Medicine I, Medical Center, University of Freiburg, Faculty of Medicine, University of Freiburg, Freiburg im Breisgau, Germany; 5 Department of Hematology and Oncology, Medical Faculty, University of Augsburg, Stenglinstr. 2, 86156 Augsburg, Germany; 6 Institute for Surgical Pathology, Medical Center - University of Freiburg, Faculty of Medicine, University of Freiburg, Freiburg im Breisgau, Germany; 7 Tumorbank Comprehensive Cancer Center Freiburg, Medical Center - University of Freiburg, Faculty of Medicine, University of Freiburg, Freiburg im Breisgau, Germany; 8 Department of Orthopedics and Trauma, Medical University of Graz, Graz, Austria; 9 Diagnostic and Research Institute of Pathology, Medical University of Graz, Graz, Austria; 10 Plastic and Reconstructive Surgery, Sir Charles Gairdner Hospital, Hospital Ave, Nedlands, WA 6009 Australia; 11 Charité – Universitätsmedizin Berlin, corporate member of Freie Universität Berlin, Humboldt-Universität zu Berlin, and Berlin Institute of Health, Institute of Pathology, Charitéplatz 1, 10117 Berlin, Germany; 12 Institute of Pathology, University Hospital Leipzig, Liebigstrasse 26, 04103 Leipzig, Germany; 13 DRK Kliniken Berlin Westend, Institute of Pathology, Spandauer Damm 130, 14050 Berlin, Germany; 14 Department of Surgery, Charité-Universitätsmedizin Berlin, Plastic and Reconstructive Surgery, Campus Virchow-Klinikum, Berlin, Germany; 15 Department of Plastic, Aesthetic and Reconstructive Surgery, Congregational Hospital Linz, Sisters of Mercy, Linz, Austria; 16 Division of Hand, Plastic and Aesthetic Surgery, University Hospital, Ludwig Maximilian University of Munich, Munich, Germany

**Keywords:** Soft tissue sarcoma, Myxoid liposarcoma, Next-generation sequencing, Targeted sequencing, Circulating tumor DNA, ctDNA, Liquid biopsy, Diagnostic biomarker, Intratumor heterogeneity

## Main text

Myxoid liposarcoma (MLS), a malignant soft-tissue tumor derived from lipocytes, is characterized by specific genetic translocations t(12;16) and t(12;22) on a background of few additional chromosomal changes [1]. About 30% of patients with localized high-grade MLS will eventually develop distant metastases [2]. Unlike other soft tissue sarcomas (STS), MLS exhibit a distinct pulmonary and extra-pulmonary metastatic pattern. Imaging for follow-up is thus extensive and requires whole-body Magnetic Resonance Imaging (MRI) or a combination of various imaging modalities [3].

Previously, we investigated the potential of circulating tumor DNA (ctDNA) to detect tumor recurrence and monitor treatment response [4]. However, due to the unique nature of each translocation and few hotspot mutations, quantification of ctDNA was technically demanding and assays were not suitable for routine diagnostics.

**Fig. 1:**
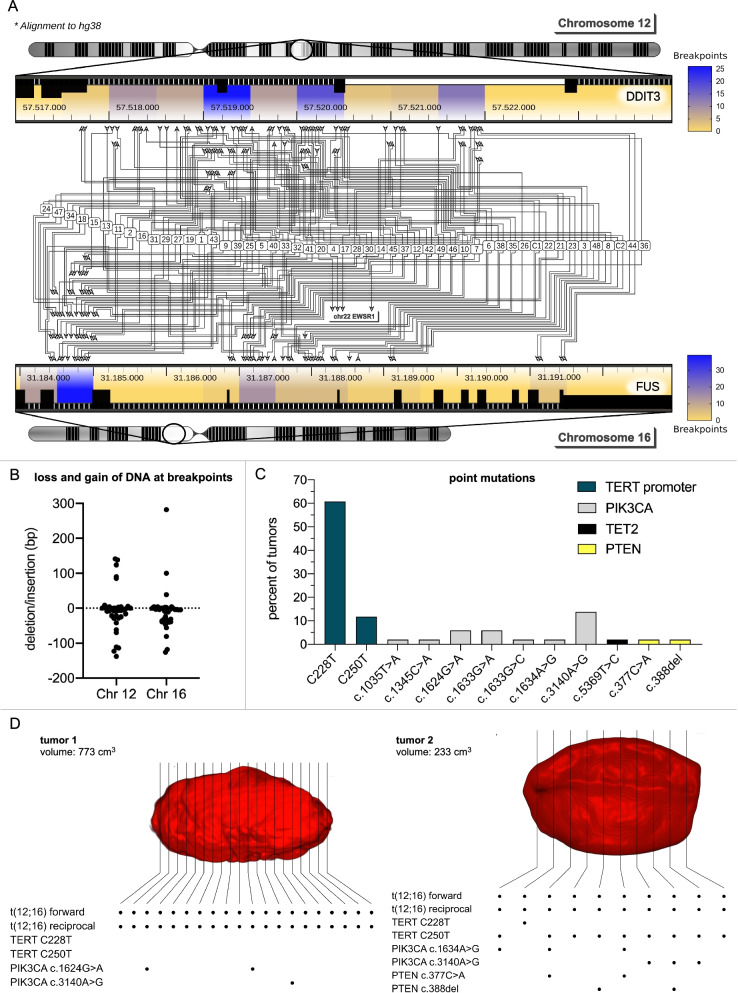
Mutational profiling of myxoid liposarcomas. **A** 51 MLS tumors and two MLS cell lines (402-91 and 1765-92) were sequenced with an MLS specific lockdown panel. Chromosomal translocations could be detected in 49/51 tumors (1 - 49) and both cell lines (C1, C2) and occurred between *DDIT3* and *FUS* in 87.7% and between *DDIT3* and *EWSR1* in 8.3%. Breakpoints clustered to several distinct regions within *DDIT3* and *FUS* but were not restricted to a single site. For 36 tumors, both breakpoints of the reciprocal translocation could be determined. Arrows depict the sites where the chromosomal breaks occurred. Areas with an increased likelihood of chromosomal breaks are colored in blue. Breakpoints were annotated to homo sapiens (human) genome assembly GRCh38 (hg38). **B** For 36 tumors, where both breakpoints could be sequenced, loss or gain of DNA during the translocation event could be determined and specifically mapped to either *DDIT3* or *FUS*. A mean loss of 7 bp (SD 64 bp) occurred on chromosome 12 (*DDIT3*) and of 11 bp (SD 73 bp) on chromosome 16 (*FUS*). There was considerable intertumor variability. Each symbol of the graph represents one tumor, with the largest gain of 282 bp on chromosome 16 and the biggest deletion of 138 bp on chromosome 12. **C** Point mutations occurred most commonly in the *TERT* promoter region (73%) and *PIK3CA* (33%). Only one tumor showed an additional point mutation in *TET2* and one tumor analyzed for intratumor heterogeneity (tumor 2 in Fig. 1 **D**) displayed two mutations in *PTEN*. *TERT* promoter mutations were all detected at the well-known hotspot locations C228T and C250T, with a prevalence of 61% and 12% of all analyzed tumors respectively. *PIK3CA* mutations occurred at well-known hotspot mutations in exon 9 (c.1624G > A, c.1633G > A, c.1633G > C, c1634A > G) and exon 20 (c.3140A > G) but also at less commonly annotated positions in exon 5 (c.1035 T > A) and exon 8 (c. 1345C > A). **D** To determine intratumor heterogeneity of MLS, 20 individual samples of tumor 1 were taken at uniform distances and 10 samples were taken from tumor 2. Each sample was analyzed separately with the *standard panel*. The individual tumor-specific breakpoints identified before (Fig. 1 A) were detectable in all samples. There were no *TERT* promoter mutations in tumor 1, however *PIK3CA* mutations were present in 3/20 samples. Two showed a hotspot mutation in exon 9 (chr3:179,218,294; c.1624G > A) and one samples had a hotspot mutation in exon 20 (chr3:179,234,297; c.3140A > G). In tumor 2, *TERT* promoter mutations were present in all samples, however nine samples contained the C250T and one sample the C228T mutations. *PIK3CA* mutations were even more diverse. 6/10 samples contained a *PIK3CA* mutation. Of these samples three showed the well-known hotspot mutation in exon 9 (chr3:179,218,304; c.1634A > G) and the remaining three the hotspot mutation in exon 20 (chr3:179,234,297; c.3140A > G). A deletion (c.388del) and a point mutation in (c.377C > A) were identified in *PTEN*. The intratumor heterogeneity of *PIK3CA, PTEN* and the *TERT* promoter point towards multiple subclones which emerge from a tumor ancestor that initially acquired the characteristic t (12;16) driver translocation. Depicted is the tumor (red), which was reconstructed from the patients’ MRI scans. The black lines depict how the tumor was sectioned for histopathologic assessment. Although the samples were taken at uniform distances throughout the tumor, the exact location of each sample within the tumor cannot be determined due to the retrospective nature of the study

To overcome these limitations, we developed targeted *next generation sequencing* (NGS) - based approaches, which allow ultrasensitive detection of MLS DNA in a routine diagnostic setting and prospective clinical trials. As standard NGS panels don’t cover common genetic alterations in MLS, we designed a lockdown panel which encompasses genes with a reported mutation frequency of at least 5%. The 36,541 base pair (bp) *standard panel* covers the introns of *DDIT3*, *FUS* and *EWS* where the t (12;16) and t (12;22) translocations occur, the *TERT* promoter region and mutation hotspots within exons from seven genes (Supplementary Fig. 1) [5–8]. Applying molecular barcodes for digital error correction [9] allowed the detection of mutations with a variant allele frequency (VAF) of 0.05% (Supplementary Fig. 2). 51 MLS tumors and two MLS cell lines (402-91 and 1765-92) were sequenced. Matched normal DNA was available for 23 tumors. Breakpoints could be identified in 49 tumors, and both cell lines. Translocations occurred in 87.7% between *DDIT3* and *FUS* and in 8.3% between *DDIT3* and *EWSR1* (Fig. 1A). No translocations were observed in matched leukocyte DNA (sensitivity of 96% and specificity of 100%).

We could determine both breakpoints of the balanced translocations in 36 of the 51 tumors and both cell lines. This allowed us to determine if deletions or insertions occurred during the translocation event. We observed a mean loss of 7 bp (SD 64 bp) on chromosome 12 (*DDIT3*) and of 11 bp (SD 73 bp) on chromosome 16 (*FUS*) with a high intertumor variability (Fig. 1B).

Point mutations were detectable in 74.5% of tumors. Mutations in the *TERT* promoter region were prevalent in 73% of tumors. Thereof the C228T mutation occurred in 61% and the C250T mutation in 12% of analyzed tumors. *PIK3CA* mutations were found in 33% of MLS samples. Besides the well-known hotspot mutations in exon 9 (c.1624G > A, c.1633G > A, c.1633G > C, c1634A > G) and exon 20 (c.3140A > G) we identified less commonly annotated mutations in exon 5 (c.1035 T > A) and exon 8 (c. 1345C > A). Only two additional genes were mutated at low frequency in our cohort, *TET2* (2%) and *PTEN* (4%) (Fig. 1C). Taken together, the lockdown panel could detect at least one mutation in 96% of all the tumor samples. Combining the breakpoints and point mutations, the panel detected an average 2.8 somatic mutations (1.7 breakpoints and 1.1 point mutations) per tumor, which can be targeted in circulating free DNA (cfDNA).

To determine the impact of tumor heterogeneity on ctDNA detection, spatially separate samples from two tumors (tumor 1 and 2 from Fig. 1A) were analyzed. Results were compared to matched white blood cells. Mutations with a VAF of at least 5 % were recorded. The two t (12;16) breakpoints were detected in all specimens. The presence of the major MLS driver translocation confirms its importance from tumor initiation to promotion. The consistency of patient individual breakpoints was previously reported for multifocal MLS [10]. In contrast, there was marked intratumor heterogeneity for *TERT promoter*, *PIK3CA* and *PTEN* mutations (Fig. 1D). This supports the hypothesis that additional mutations seem to occur secondarily and evolve within different tumor subclones [11]. Thus, tracking of breakpoint fragments in cfDNA promises detection of the primary tumor and all its potential metastases. In contrast, point mutations identified in the primary tumor might or might not be present in its metastases, depending of their clones of origin.

**Fig. 2:**
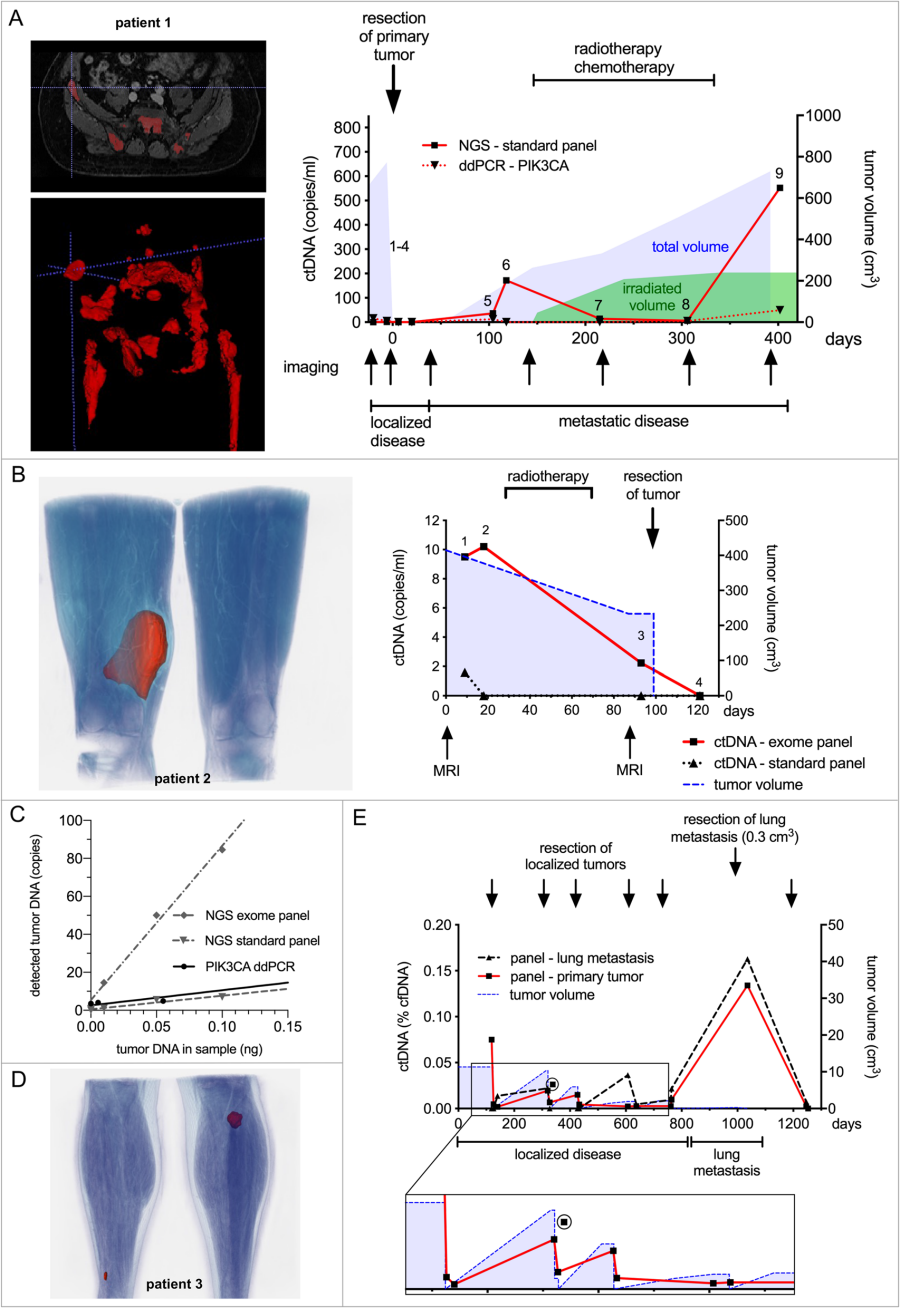
Quantification of ctDNA in patients’ plasma samples. **A** Quantification of ctDNA in 9 plasma samples of patient 1 collected during 2 years of treatment. ctDNA was determined by NGS (*standard panel)* and *PIK3CA* mutations (c.1624G > A and c.3140A > G) were additionally quantified by ddPCR. He initially presented with a localized MLS of the thigh which was completely resected. Soon after, he developed metastatic disease with predominantly osseous lesions. He then received radiotherapy of bone metastasis and several courses of chemotherapy. Repeated imaging during follow-up showed numerous new skeletal lesions and the patient again received radiotherapy to selected metastasis. He succumbed to his disease 1.5 years after removal of the primary tumor. ctDNA increased to 172 copies/ml (sample 6) when metastatic disease was detected and decreased during radio/chemotherapy to 14 copies/ml (sample 7) and 7 copies/ml (sample 8). There was a rapid incline in ctDNA when multiple new metastases were detected (sample 9: 552 copies/ml). Standard imaging, which reflects the total mass of viable and necrotic tumor cells, showed a steady increase (blue area). The irradiated tumor volume is depicted as a surrogate marker for the necrotic tumor mass (green area). t (12;16) ctDNA levels were higher than *PIK3CA* ctDNA concentrations. This most likely reflect intertumor heterogeneity with only a fraction of metastases carrying *PIK3CA* mutations (Fig. 1 D and Supplementary Fig. 4). **B** Additional target mutations from exome sequencing increase sensitivity of ctDNA detection. Tumor 2 was subjected to exome sequencing to identify additional target mutations. Together with breakpoints and mutations from the *standard panel*, a 7320 bp hybrid e*xome panel* targeting 15 genomic regions was designed. ctDNA in plasma obtained during treatment was determined by the *standard* and *exome* panel. He initially received neo-adjuvant radiotherapy to an MLS of his right thigh and subsequently the tumor was completely resected. Two plasma samples were collected prior to commencement of radiotherapy, a third sample before surgery and a fourth sample after tumor resection. ctDNA quantified by the exome panel (red line) was present in similar amounts at the two time points before treatment, declined after radiotherapy and was not detectable after tumor resection. The *standard panel* (dashed black line) could detect ctDNA only in the first sample, showing reduced sensitivity compared to the *exome panel*. The blue area represents the tumor volume as calculated from the MRI scans. **C** Comparison of different assays in detecting MLS tumor-DNA. Dilution series of MLS tumor-DNA from two tumors (patient 2 and 3) in matched normal DNA were analyzed by ddPCR (*PIK3CA* mutations p.N345K, c.1035 T > A and p.E545G, c.1634A > G), the NGS *standard panel* and respective *exome panels*. Depicted are mean values and linear regression of *n* = 2 tumors for ddPCR, n = 2 for the *standard panel* and n = 2 for the *exome panels*. We observed a similar performance for ddPCR and the *standard panel,* whereas detection of tumor-DNA with *exome panels* was clearly superior. **D** Patient 3 presented with two small localized tumors of his legs (red) after numerous prior resections at another hospital. The tumors were completely resected, but he repeatedly developed local recurrences at both locations in the following years. **E** These recurrences were subsequently resected at four consecutive operations before a small lung metastasis (0.3 cm^3^) was detected and subsequently removed. *Exome panels* were obtained from sequencing one of the primary lesions and the lung metastasis. ctDNA was subsequently quantified with both panels in 15 plasma samples obtained during the course of his treatment. During multifocal localized disease, ctDNA values undulated at low concentrations depending on the presence of viable tumor tissue. The *exome panel* from the primary tumor best reflected the clinical course (enlarged image section). There was one outlier (circle). Despite complete tumor resections, ctDNA values never reached the baseline indicating MRD. In contrast the plasma sample obtained shortly before resection of the lung metastasis showed markedly increased ctDNA with a decline after its resection. The red line represents ctDNA values measured by the *exome panel* from the primary lesion and the dashed line ctDNA measured by the *exome panel* obtained from the lung metastasis. The blue area depicts the tumor volume

The assay was subsequently employed to quantify ctDNA in plasma samples of MLS patients. Nine plasma samples, collected during the 2-year treatment of patient 1 (see Fig. 1D) with a localized MLS who later developed metastatic disease were analyzed with the *standard panel*. The two *PIK3CA* mutations (c.1624G > A and c.3140A > G) were additionally quantified by droplet digital PCR. ctDNA levels decreased after tumor resection and increased when metastatic disease was detected. We observed a decline in ctDNA when radio/chemotherapy was initiated. However, with increasing tumor burden, concentrations rose again after several months (Fig. 2A and Supplementary Fig. 3 A). Serial ctDNA testing promises monitoring of treatment response in metastatic MLS. It might be especially beneficial for patients treated with immunotherapeutic agents, that challenge established imaging-based response assessment criteria [12, 13].

From the two *PIK3CA* mutations identified in the primary tumor, only c.3140A > G ctDNA correlated with t (12;16) ctDNA. This indicates that most metastases originated from a clone in the primary tumor, which harbored this mutation (Supplementary Fig. 4). As new treatment opportunities which target *PIK3CA* are only effective in *PIK3CA* mutated cells [14], liquid biopsy may enable us to identify patients with druggable metastases without the need of repeated biopsies.

Further mutations obtained by exome sequencing of individual tumors were added to the mutations already identified by the *standard panel* to lower the limit of detection (LoD) (Supplementary Fig. 5). These hybrid panels (*exome panels*) allowed us to monitor ctDNA of localized tumors as exemplified in the following scenario. Patient 2 received neoadjuvant radiotherapy and subsequently complete resection (tumor necrosis rate > 90%) of a localized MLS. Two plasma samples obtained at an interval of 9 days before initiation of radiotherapy showed similar concentrations of ctDNA (Fig. 2B). The third specimen obtained after radiotherapy demonstrated markedly decreased ctDNA. No ctDNA was detectable in the fourth sample collected after surgery. Analysis with the *standard panel* detected ctDNA in the initial plasma sample only. Calculating relative amounts, the fraction of ctDNA was between 0% and 0.05% (Supplementary Fig. 3 B). Detection of these minute amounts in limited plasma samples requires extremely sensitive assays. This was accomplished by *exome panels,* which target multiple mutations simultaneously, thus detecting more mutant copies in the same amounts of tumor DNA than ddPCR and the *standard panel* (Fig. 2C and Supplementary Fig. 5).

The impact of intertumor heterogeneity on ctDNA quantification was assessed in a patient who was initially presented with multifocal disease of his legs after previous resections at another hospital (Fig. 2D). In the course of his treatment, he suffered from four local recurrences before a small lung metastasis (0.3 cm^3^) was identified and resected. Exome sequencing was conducted from the initial leg tumor and the lung metastasis. The *exome panels* targeted 22 and 17 genomic regions respectively, and 6 mutations were identical in both panels. ctDNA in 15 plasma samples was quantified with both panels. During localized disease ctDNA fluctuated depending on the amount of viable tumor mass. The panel from the primary lesion performed superior during localized disease. The small lung metastasis however, led to markedly increased ctDNA concentrations which again decreased after resection. Both panels performed similarly during metastatic disease (Fig. 2E). Comparatively high ctDNA concentrations of lung metastases in MLS might enable detection of recurrence earlier by liquid biopsy than with imaging-based approaches [4, 15].

## Conclusions

In this study, we present an approach for ctDNA monitoring in MLS patients in a routine diagnostic setting using a disease and patient-specific hybrid capture NGS technique. Quantification of ctDNA on the basis of cancer genomic profiling could help to predict tumor recurrence, and monitor tumor heterogeneity and treatment response in metastatic disease with minimal invasiveness and at affordable cost. The assay can easily be adapted to other translocation driven tumors, e.g. synovial sarcomas. Given our promising results, the methods we have described warrant investigations in prospective trials with larger cohorts, so they can timely be translated into clinical practice.

## Supplementary Information

**Additional file 1: Supplementary Figure 1.** Specifications of standard lockdown panel for MLS. **Supplementary Figure 2.** Evaluation of limit of detection (LoD). **Supplementary Figure 3.** Comparison of absolute and relative ctDNA quantification. **Supplementary Figure 4**. Impact of tumor heterogeneity on ctDNA detection. **Supplementary Figure 5.** Additional target mutations from exome sequencing increase sensitivity of tumor DNA detection. **Supplementary Figure 6.** Determination of sensitivity and specificity of standard panel.

**Additional file 2:** Materials and Methods.

## Data Availability

The datasets used and/or analyzed during the current study are available from the corresponding author on reasonable request.

## Abbreviations

MLS: Myxoid liposarcoma
STS: Soft tissue sarcoma
ctDNA: Circulating tumor DNA
cfDNA: Circulating free DNA
NGS: Next generation sequencing
bp: Base pair
FFPE: Formalin-Fixed Paraffin-Embedded tissue
hg38: *Homo sapiens* (human) genome assembly GRCh38
ddPCR: Droplet digital PCR
VAF: Variant allele frequency
MRI: Magnetic Resonance Imaging
SD: Standard Deviation
LoD: Limit of Detection
