# Supplementary material for: Targeted next-generation sequencing of circulating free DNA enables non-invasive tumor detection in myxoid liposarcomas
Source: Mol Cancer. 2022 Feb 14;21:50. doi: 10.1186/s12943-022-01523-x (PMC8842903; doi:10.1186/s12943-022-01523-x)

Supplementary Figure 1

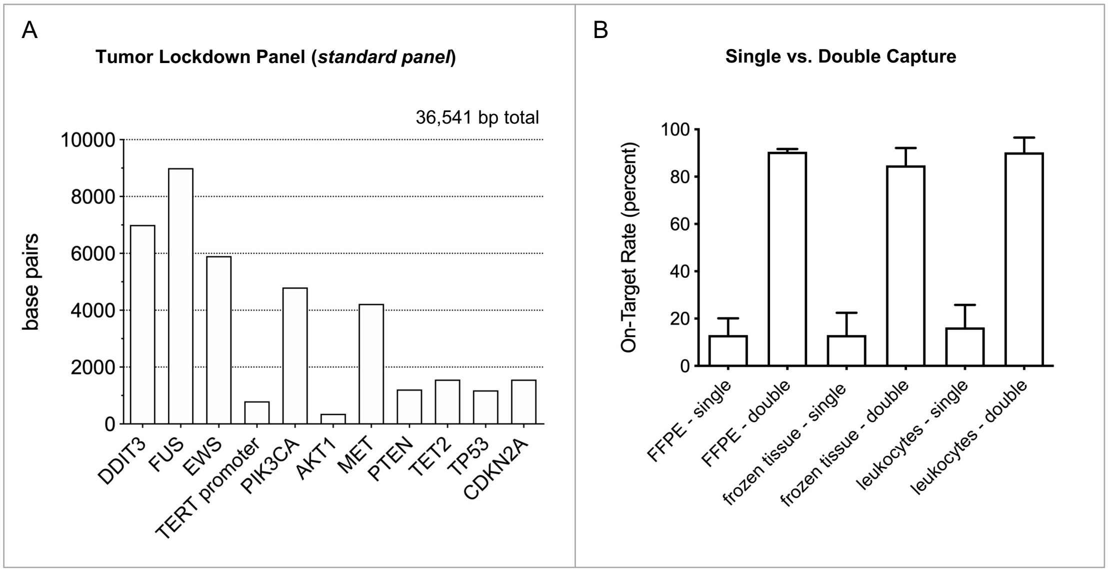

Supplementary Figure 2

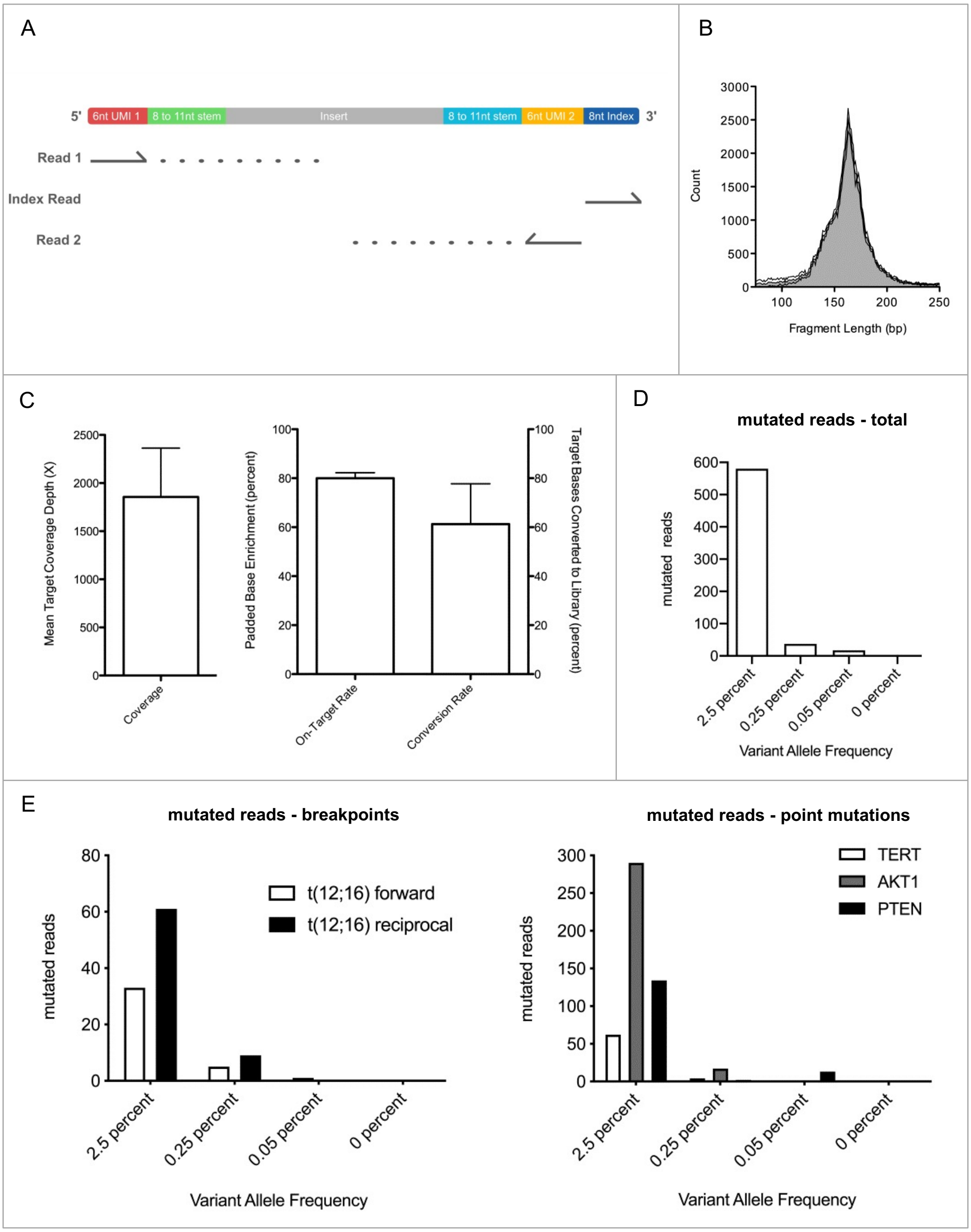

Supplementary Figure 3

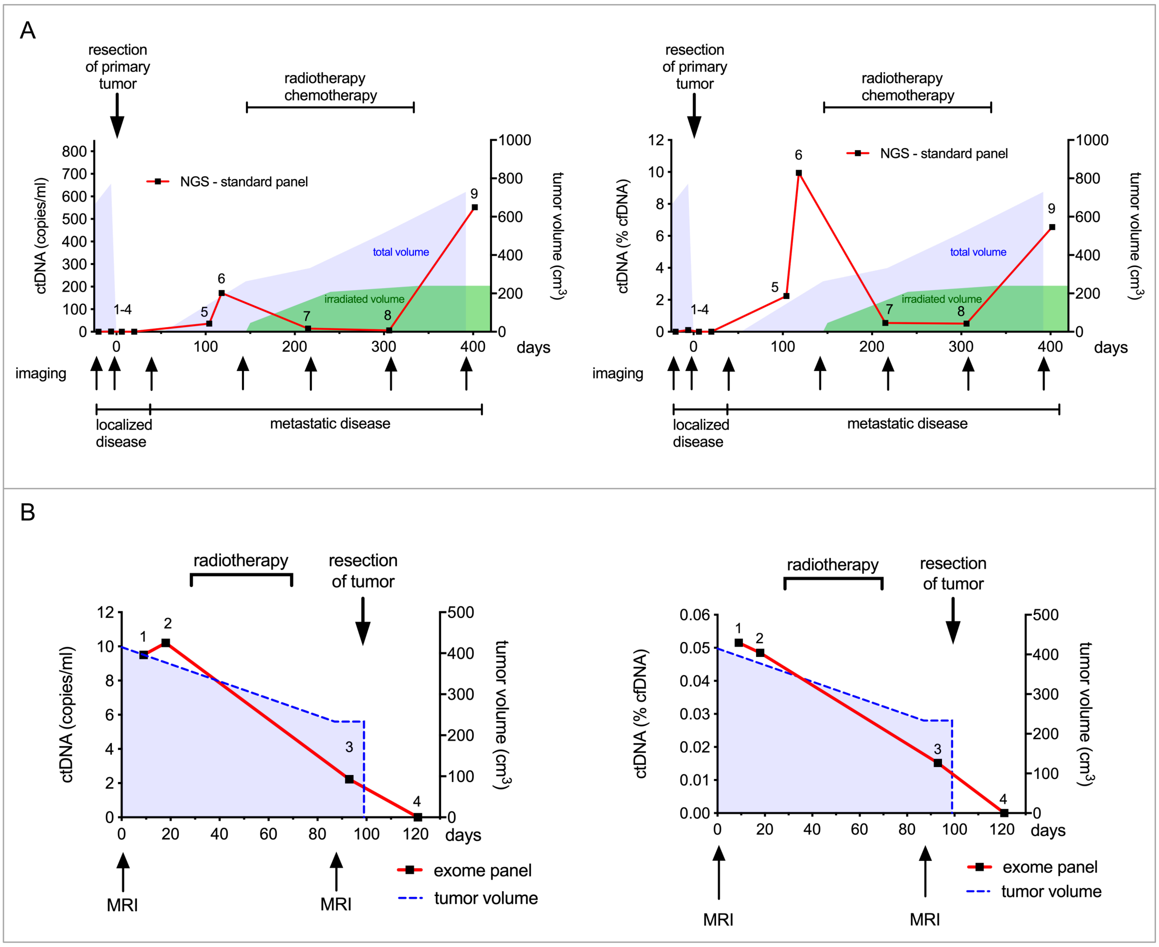

Supplementary Figure 4

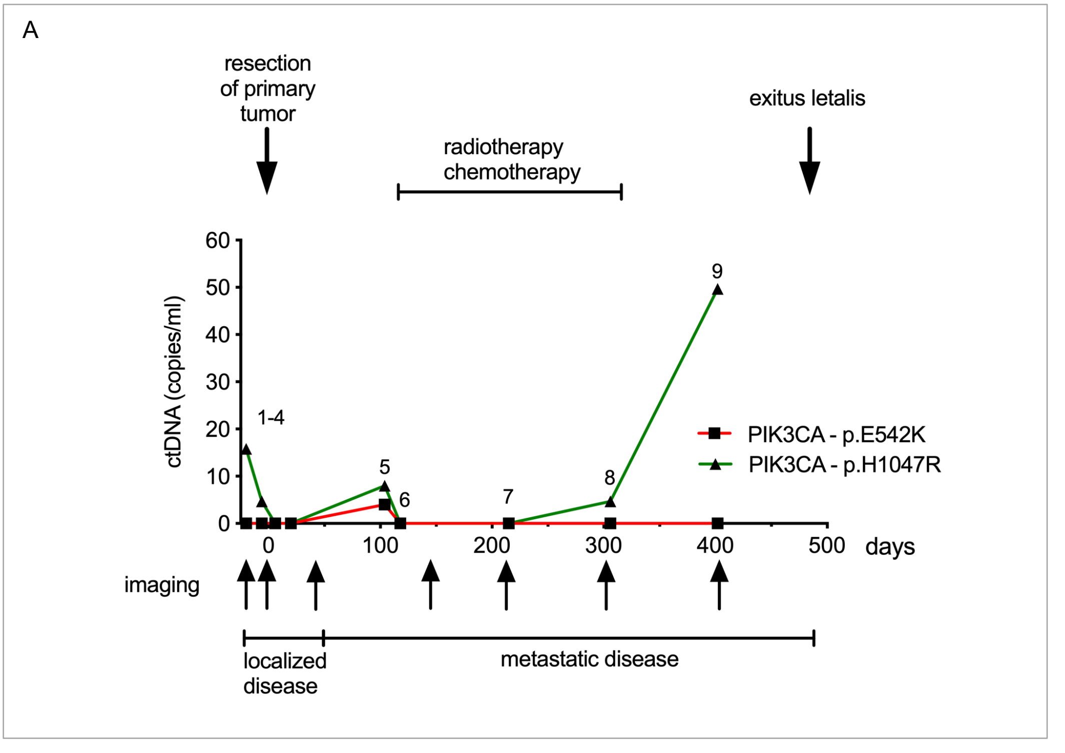

Supplementary Figure 5

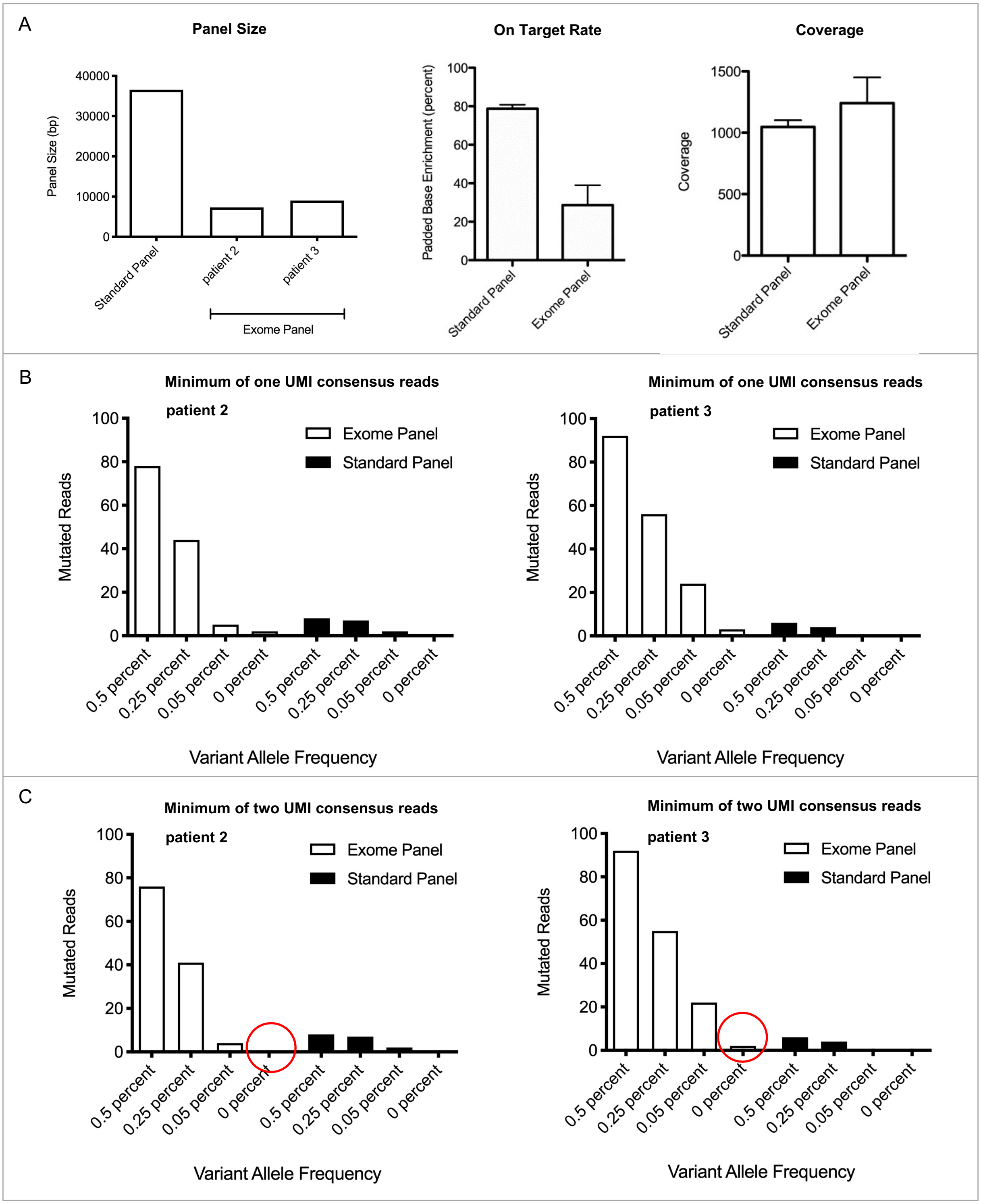

Supplementary Figure 6

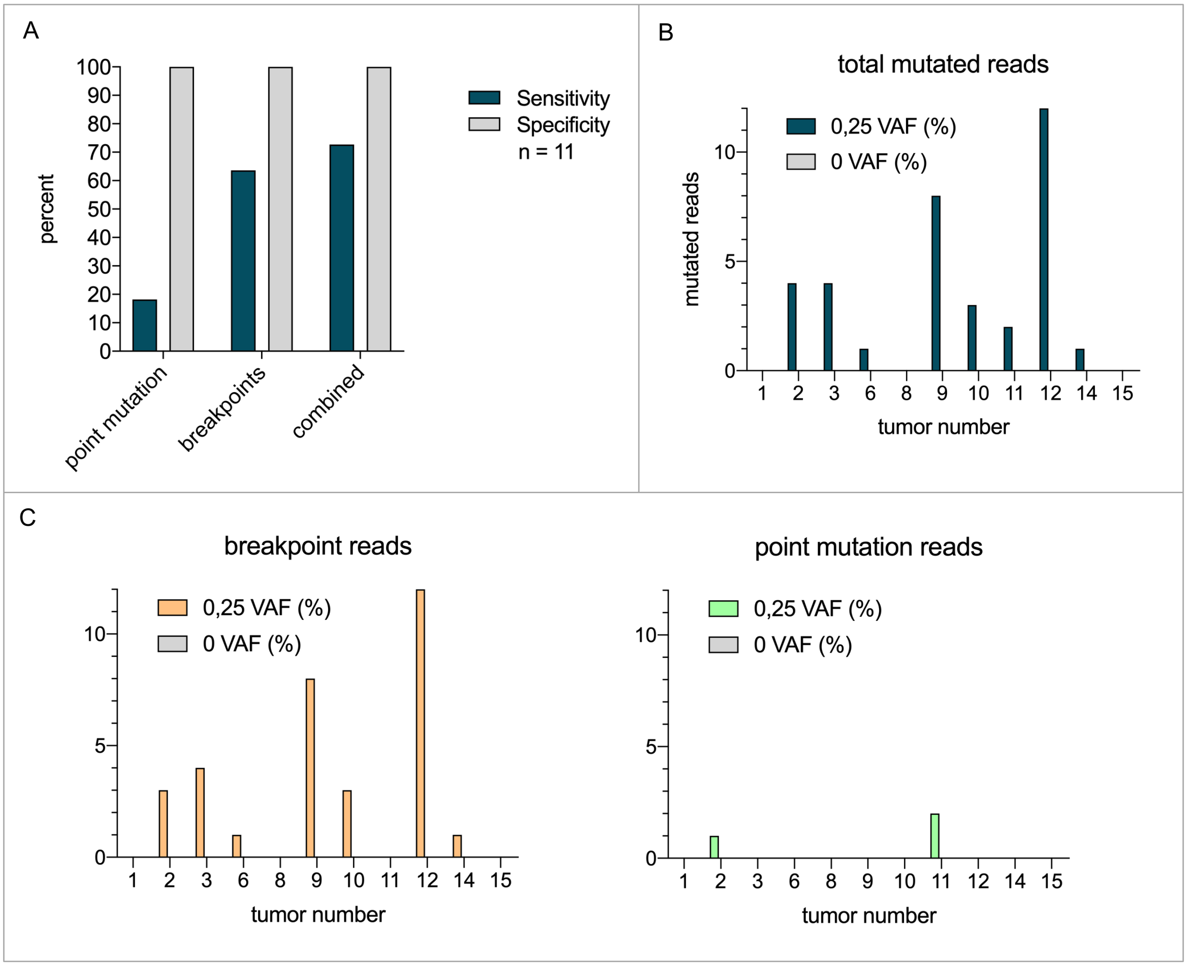

Supplement: Supplementary file 1 — Additional file 1: Supplementary Figure 1. Specifications of standard lockdown panel for MLS. Supplementary Figure 2. Evaluation of limit of detection (LoD). Supplementary Figure 3. Comparison of absolute and relative ctDNA quantification. Supplementary Figure 4. Impact of tumor heterogeneity on ctDNA detection. Supplementary Figure 5. Additional target mutations from exome sequencing increase sensitivity of tumor DNA detection. Supplementary Figure 6. Determination of sensitivity and specificity of standard panel. [file 12943_2022_1523_MOESM1_ESM.pdf]
