# Supplementary material for: Targeted next-generation sequencing of circulating free DNA enables non-invasive tumor detection in myxoid liposarcomas
Source: Mol Cancer. 2022 Feb 14;21:50. doi: 10.1186/s12943-022-01523-x (PMC8842903; doi:10.1186/s12943-022-01523-x)
Supplement: Supplementary file 2 — Additional file 2: Materials and Methods. [file 12943_2022_1523_MOESM2_ESM.docx]

**Title: Targeted next-generation sequencing of circulating free DNA enables non-invasive tumor detection in myxoid liposarcomas**

**Authors:**

Eisenhardt AE, Schmid A, Esser J, Brugger Z, Lausch U, Kiefer J, Braig M, Runkel A, Wehrle J, Claus R, Bronsert P, Leithner A, Liegl-Atzwanger B, Zeller J, Papini R, von Laffert M, Pfitzner BM, Koulaxouzidis G, Giunta RE, Eisenhardt SU, Braig D

# Materials and Methods

**Study population**

Samples in this study were obtained from 51 MLS patients in total. The cohort contained 17 patients who were treated at the Comprehensive Cancer Center Freiburg (CCCF) (Freiburg, Germany), 27 samples were from the Institute of Pathology, Charité (Berlin, Germany) which had been processed and prepared by the Central Biobank Charité (ZeBanC) and 7 samples were obtained from the Diagnostic and Research Institute of Pathology, Medical University of Graz (Graz, Austria) in cooperation with the Biobank of the Medical University of Graz.

**Blood and tissue sampling**

All blood samples were collected by puncture of the antecubital vein without tourniquet through a 20-gauge needle. The first 3 ml of blood were discarded. Each 9 ml of whole blood were collected in K_2_EDTA (1,6 mg EDTA / ml blood) tubes (Sarstedt AG & Co, Nümbrecht, Germany). Blood was processed within 2 h after blood withdrawal. Blood samples were double centrifuged for 15 min at 2,500 g at 22 °C. Plasma aliquots were stored in cryotubes (FluidX) at -80 °C before use.

**Isolation of DNA from native and FFPE tissue**

DNA was extracted from tumor native tissue according to the manufacturer’s instructions, DNeasy Blood and Tissue Kit (Qiagen, Hilden, Germany). While DNA from formalin-fixed paraffin-embedded (FFPE) was extracted using the FFPE Qiagen Extraction Kit (QIAmp DNA FFPE Tissue Kit). Approximately 8 sections 5-10µm, were digested with Proteinase K at 56° C for 3 days. An elution in RNase free water, volume of 80µl was used.

**Isolation of DNA from blood/leukocytes**

DNA was extracted from blood leukocytes with the DNeasy Blood and Tissue Kit (Qiagen, Hilden, Germany). The DNA was eluted in a volume of 200 µl RNase free water.

**Cell-free DNA isolation**

Cell-free DNA was extracted from 4 ml to 5 ml of plasma using the QIAamp Circulating Nucleic Acid Kit (Qiagen, Hilden, Germany) according to the manufacturer’s protocol. An elution in AVE buffer, volume of 30 µl was used. Purified cfDNA was stored in Lo Bind DNA tubes (Eppendorf, Hamburg, Germany) at -20 °C.

**Quantification of cell-free DNA**

Quantity of cfDNA in plasma was determined by Qubit 3.0 Fluorometer, dsDNA HS Assay Kit (Invitrogen, Carlsbad, CA).

**Library preparation for tumor, leukocyte and cfDNA.**

DNA from tumor tissue (tumor native or FFPE) and matched control from whole blood, were prepared for sequencing by generating libraries using NEB kit NEBNext Ultra II FS DNA Library Prep Kit for Illumina (New England Biolabs, Ipswich, MA, USA). DNA was fragmented to optimal length (approximately 150 bp) by incubation with 1 µl NEBNext Ultra II FS Enzyme for 25 min at 37° C. 60 ng DNA input was used, followed by 8 polymerase chain reaction (PCR) cycles.

Libraries from cfDNA were generated according to the manufacturer’s manual Takara SMARter ThruPLEX Tag-Seq 48S Kit (Takara Bio In., Kusatsu, Shiga, Japan). 10 ng input of cfDNA was used and adapters were ligated to attach unique molecular identifiers (UMI) to each side of the DNA fragment with single indices (Supplementary Figure 2 A).

**Target enrichment for tumor and leukocyte libraries**

A tumor lockdown panel (*standard panel*) was designed, which encompassed breakpoint regions and exons of genes that are hotspot regions in MLS (1–4), (Supplementary Figure 1 A).

IDT xGen Panels comprising biotin probes were used to hybridize target regions. Following hybridization, target regions were pulled down with streptavidin-coated magnetic beads. The protocol was performed according to the manufacturer’s instructions, xGen Hybridization and Wash Kit (Integrated DNA Technologies, Coralville, IA, USA). Following hybridization and PCR post capture samples were size selected using Agencourt AMPure XP beads (Beckman Coulter). Double capture was performed with libraries of 100 – 250 ng pooled to total 500 ng – 1 µg. Both captures were incubated for 4 h at 65 °C (Supplementary Figure 1 B).

**Target enrichment for cfDNA libraries**

Target enrichment with cfDNA libraries was performed with custom x-Gen lockdown pool probes from the *standard panel* described above or patient-specific *exome panels*. These were designed based on the mutations identified in exome sequencing (CeGaT GmbH, Tübingen, Germany) performed on three tumors (Integrated DNA Technology, Coralville, IA, USA).

Libraries were purified with Agencourt AMPure XP beads (Beckman Coulter). DNA concentration of the libraries were measured with Qubit (Invitrogen, Carlsbad, CA) and equal amounts of each library were pooled (between 100 – 250 ng per library) for hybridization according to the xGen Hybidization and Wash Kit (Integrated DNA Technology, Coralville, IA, USA) protocol adapted in order to perform double capture. The first capture was incubated at 65 °C for 4 h, followed by post capture PCR with 16 cycles. A second capture was performed with the same probes also at 65 °C for 4 h, and 9 cycles post capture PCR. The length of the cleaned up captured libraries were then measured by Tape Station Agilent D500 (Agilent, Santa Clara, CA, USA). For accurate DNA concentration, libraries were measured with qPCR LightCycler 480 System (Roche), using the NEBNext Library Quant Kit for Illumina (New England Biolabs).

The desired amount of the libraries was then calculated and sequenced using a MiSeq system with paired end reads, MiSeq V2 300 cycles (Illumina Inc. San Diego, CA, USA).

**ddPCR**

Quantification of PIK3CA hotspot mutations was performed with the QX100 Droplet Digital PCR System (BioRad). Commercially available assays were used (BioRad; UniqueAssayID: HsaMDV2516778 for p.N345K, c.1035T>A and assay ID: dHsaMDV2510544 for p.E545G, c.1634A>G)

Samples were prepared according to the manufacturer’s recommendations. The PCR was performed with the following cycling conditions: The enzyme activation took place at 95° C for 10 min. It was followed by 48 cycles of denaturation at 94° C for 30 s, annealing at 55° C for 60 s and extension at 62° C for 60 s. The enzyme deactivation took place at 98°C for 10 min. Fluorescence within each droplet was subsequently determined by the QX100 droplet reader and data analyzed with QuantaSoft Analysis Software (BioRad).

**Bioinformatics**

The sequence reads of tumor tissue samples (tumor native or FFPE) were uploaded to Illumina BaseSpace (Illumina Inc., San Diego, CA, USA) and aligned to human genome (hg38). Marking for duplicates and calling somatic mutations including single nucleotide variants and short indels were detected using Dragen Somatic Pipeline (BaseSpace, Illumina Inc., San Diego, CA, USA). Patient matched blood-samples were analyzed simultaneously in order to eliminate germline variants.

For structural variant analysis the sequence reads were uploaded to the Galaxy web-platform, aligned to human genome (hg38) using BWA-MEM algorithm *(5)* and marked for duplicates using SAMtools software package (http://www.htslib.org/). Afterwards, *de novo* identification of breakpoints was performed with FACTERA *(6)*. The resulting breakpoints were verified by creating an individual custom fasta sequence for each patient and by realigning the sequence reads. Structural variants in cfDNA samples containing UMIs were detected using the MAGERI computational pipeline *(7)*. In this process, UMIs were extracted from the sequence reads and subsequently, consensus reads were assembled and aligned to previously described custom fasta sequences.

To reduce the ratio of false positives each alteration was manually reassessed with the Integrative Genomics Viewer (IGV) *(8)*. In order to obtain general information about the performance of the panels the sequence reads were further processed with the SAMtools software package (http://www.htslib.org/) and Illumina Dragen Enrichment (BaseSpace, Illumina Inc., San Diego, CA, USA). All events were documented in GRCh38 assembly.

For analysis of single nucleotide variants, sequence reads of cfDNA samples containing UMIs were uploaded to the Curio Genomics web-platform (https://curiogenomics.com) and aligned to human genome (hg38) using Bowtie2. Ensuing, UMI-family consensus reads were called and somatic mutations including single nucleotide variants were discovered with the Curio Genomics built-in analysis pipeline. For mutation calling, a minimum of 3 reads per UMI with at least 60 percent consensus reads were required. Paired-end reads were included in the analysis pipeline.

To calculate the relative fraction of ctDNA, the mutated reads at each position are recorded and subsequently divided by the total coverage at the same position. For relative quantification of breakpoint reads, the adjacent bases of DDIT3/FUS serve as the reference to determine the coverage across the breakpoint region.

**Analysis of assay performance**

The goal in assay development is to develop robust methods that can tolerate minor changes and that will ultimately help sustain a higher level of productivity and efficiency (9). The Blood Profiling Atlas Consortium (BloodPAC) has recently published generic protocols for the analytical validation of Next-Generation Sequencing-Based ctDNA assays (10). We have adapted their extensive recommendations to perform an early determination of assay robustness and analytical validation for the following factors:

1. Evaluation of blood collection tubes and preanalytical considerations: Standard EDTA blood collection tubes and “Cell-Free DNA Collection Tubes” from Roche have been evaluated prior to the start of analytical assay validation. Differences in storage time and sample shipping have been determined and were published by our group recently (11).

2. Evaluation for analytical accuracy: Analytical accuracy of breakpoint sequencing in tumor tissue was assessed by method comparison. In addition to NGS sequencing with the *standard panel*, the breakpoints of both cell lines and seven tumors were validated by long-range PCR amplification and Sanger Sequencing as previously described (12). Both methods revealed identical sequence motifs at the tumor breakpoints.

3. Evaluation of a reference interval: The reference interval refers to the range of sequence variation and known polymorphisms that are found in the normal population that are detectable by the assay. Besides the 51 tumor samples, we sequenced 23 matched-normal leukocyte samples with the standard panel, which have been processed using the same conditions. No t(12;16) or t(12;22) translocations, *TERT promoter mutations* or any of the ten hotspot mutations identified in *PIK3CA, PTEN* and *TET2* (Figure 1 C) were detected in the respective reference samples. For *in silico* evaluation of known polymorphisms, the pathogenicity of these variants was additionally confirmed on GnomAD (13).

4. Evaluation of limit of blank (LOB): The false positive rate was established using samples with cfDNA from healthy donors and matched normal samples from leukocyte DNA.

For the *standard panel,* a separate cfDNA only sample was run in the dilution series of MLS 402-91 cell-line DNA (Supplementary Figure 2) and matched leukocyte-only DNA was evaluated in the dilution series of tumor 2 and tumor 3 (Supplementary Figure 5). Additional nine matched normal leukocyte samples were analyzed to determine the specificity of the assay (Supplementary Figure 6). There were no false positive events detectable in all samples.

For the *exome panels*, leukocyte-only samples were analyzed as part of the dilution series of tumor 2 and 3. The false-positive rate was reduced by including a minimum of two UMI consensus reads for variant calling. There were no false positive events in the exome panel for patient 2, and only one false positive event for patient 3 (Supplementary Figure 5).

5. Evaluation of limit of detection (LoD): LoD is defined as the lowest allelic frequency that can be detected. For evaluation of the *standard panel,* a dilution series of MLS 402-91 cell-line DNA in cfDNA of a healthy donor was performed. Both t(12;16) breakpoints and single nucleotide variants in the *TERT promoter, AKT* and *PTEN* could be tracked at variant allele frequencies of 2.5, 0.25, 0.05 and 0 percent. Mutant reads could still be detected at the lowest VAF of 0.05 percent (Supplementary Figure 2). We additionally analyzed a dilution series of tumor DNA in matched white blood cell DNA from patient 2 and 3 with the same VAF. The lowest VAF of 0.05 percent could be detected in patient 2, but not in patient 3. A VAF of 0.25 percent was detectable in both patients (Supplementary Figure 5). For evaluation of the *exome panels*, the same two dilution series were analyzed with the respective *exome panels*. Here, even the lowest VAF of 0.05 percent was reliably detectable in both patients (Supplementary Figure 5).

The efficiency of the panels in detecting fusions or point mutations at low variant allele frequencies is about equal (fusions/point mutations: 1.08, SD 0.85, n=8 samples).

6. Evaluation of sensitivity and specificity: Tumor mutational profiling with the *standard panel* revealed t(12;16) and t(12;22) translocations in 49/51 tumors. No breakpoint reads were observed in matched leukocyte DNA. Only fusion positive tumors, as determined by FISH, were included in the study. This translates to a sensitivity of 96% and specificity of 100%.

In addition, the sensitivity and specificity of the *standard panel* to detect limited tumor DNA amounts was evaluated. Tumor DNA of 11 patients was mixed in matched leukocyte DNA (10 ng total) at a calculated VAF of 0.25 percent. Tumor DNA was detectable in 8/11 cases (Sensitivity: 73 percent). There were 0 false positive events (Specificity: 100 percent). If only breakpoints were included in the analysis, the sensitivity of the assay was 64 percent (7/11 cases). Considering point mutations only, the sensitivity was 18 percent (2/11 cases) (Supplementary Figure 6).

**Volume rendering of tumors**

Tumor volume analysis was performed on image data from MRI and Computer Tomography (CT) acquisitions. The tumors were delineated in the images using HOROS (Horosproject.org). Volume rendering and visualization of the tumors was done using EnSight 10.2 (ANSYS, Cannonsburg, USA).(*(14)*).

**References**

1. Koelsche C, Renner M, Hartmann W, Brandt R, Lehner B, Waldburger N, et al. TERT promoter hotspot mutations are recurrent in myxoid liposarcomas but rare in other soft tissue sarcoma entities. J Exp Clin Cancer Res. 2014;33:33.

2. Demicco EG, Torres KE, Ghadimi M, Colombo C, Bolshakov S, Hoffman A, et al. Involvement of the PI3K/Akt Pathway in Myxoid/Round Cell Liposarcoma. Mod Pathol. 2012;25:212–21.

3. Powers MP, Wang W-L, Hernandez VS, Patel KS, Lev DC, Lazar AJ, et al. Detection of myxoid liposarcoma-associated FUS-DDIT3 rearrangement variants including a newly identified breakpoint using an optimized RT-PCR assay. Mod Pathol. 2010;23:1307–15.

4. Movva S, Wen W, Chen W, Millis SZ, Gatalica Z, Reddy S, et al. Multi-platform profiling of over 2000 sarcomas: identification of biomarkers and novel therapeutic targets. Oncotarget. 2015;6:12234–47.

5. Afgan E, Baker D, Batut B, van den Beek M, Bouvier D, Cech M, et al. The Galaxy platform for accessible, reproducible and collaborative biomedical analyses: 2018 update. Nucleic Acids Res. 2018;46:W537–44.

6. Newman AM, Bratman SV, Stehr H, Lee LJ, Liu CL, Diehn M, et al. FACTERA: a practical method for the discovery of genomic rearrangements at breakpoint resolution. Bioinformatics. 2014;30:3390–3.

7. Shugay M, Zaretsky AR, Shagin DA, Shagina IA, Volchenkov IA, Shelenkov AA, et al. MAGERI: Computational pipeline for molecular-barcoded targeted resequencing. PLoS Comput Biol. 2017;13:e1005480.

8. Robinson JT, Thorvaldsdóttir H, Winckler W, Guttman M, Lander ES, Getz G, et al. Integrative Genomics Viewer. Nat Biotechnol. 2011;29:24–6.

9. Cowan KJ. On assay robustness: the importance of early determination and science-driven decision-making. Bioanalysis. 2013;5:1317–9.

10. Godsey JH, Silvestro A, Barrett JC, Bramlett K, Chudova D, Deras I, et al. Generic Protocols for the Analytical Validation of Next-Generation Sequencing-Based ctDNA Assays: A Joint Consensus Recommendation of the BloodPAC’s Analytical Variables Working Group. Clinical Chemistry. 2020;66:1156–66.

11. Braig D, Becherer C, Bickert C, Braig M, Claus R, Eisenhardt AE, et al. Genotyping of circulating cell-free DNA enables noninvasive tumor detection in myxoid liposarcomas. Int J Cancer. 2019;145:1148–61.

12. Xiang H, Wang J, Hisaoka M, Zhu X. Characteristic sequence motifs located at the genomic breakpoints of the translocation t(12;16) and t(12;22) in myxoid liposarcoma. Pathology. 2008;40:547–52.

13. gnomAD: https://gnomad.broadinstitute.org/

14. Yushkevich PA, Piven J, Hazlett HC, Smith RG, Ho S, Gee JC, et al. User-guided 3D active contour segmentation of anatomical structures: significantly improved efficiency and reliability. Neuroimage. 2006;31:1116–28.
